# Prevalence of Valproate Prescription in Males 18– 55 Years

**DOI:** 10.1192/bjo.2025.10569

**Published:** 2025-06-20

**Authors:** Kareem Darweesh, Sarah Jones

**Affiliations:** GMMH, Manchester, United Kingdom

## Abstract

**Aims:** To understand the number of men of childbearing age that are prescribed valproate on the inpatient wards in GMMH.

The number of men who are advised about the reproductive safety of valproate.

The number of men who have consented to valproate following discussion around the reproductive issues and have a risk assessment completed.

**Methods:** Attaining patients’ details was initially done by communicating with lead pharmacists in every ward, to help with the data collection, in line with collecting data available also on PARIS system, EPMA the electronic prescribing system, having the information about patients’ prescriptions, doses, ward, ward capacity, consent, and start date of the prescription, has been done through patient notes on PARIS system, going through the notes for the last 20 years to ensure having all the information needed, and searching for every needed information with more than keyword, for example:

Deep searching for the notes for the valproic acid by the word (Valproate, Valproic, Epilim, Depakote, Epistena, Convulex, Sodium Valproate).

Deep searching for the notes if the patient was informed on Contraception and potential teratogenic harm by the words (Contraception, Birth, Teratogenic, PREVENT, informed).

**Results:** A considerable number and percentage of inpatient males, around 16%, aged between 18 and 55 years, are prescribed valproate.

The main rationale/indication behind the prescribing was as a mood stabilizing agent.

There was no evidence on the notes, or forms on the system, that any of the patients were advised regarding its reproductive side effects and long-term safety.

There was no evidence on the notes, or forms on the system, that any of the patients discussed the reproductive side effects or filled out a risk form for valproate before taking it.

**Conclusion:** The results show that the current prescribing of valproate for male inpatients within GMMH on 8 May 2024 does not meet the MHRA guidance.

There was no documented evidence that any of the males prescribed valproate were informed of the risks associated with reproductive safety or had received the patient information leaflet or had a risk assessment completed or had a review date set.

Given the current and updated MHRA guidance, as of Jan 2024, GMMH needs to implement measures at pace to improve the prescribing practices associated with valproate in males.

